# The digital divide among U.S. Hospitals delivering health services to populations with high social disparities

**DOI:** 10.3389/fdgth.2026.1822230

**Published:** 2026-07-30

**Authors:** Anne Snowdon, Abdulkadir Hussein, Alexandra Wright, Tim Storey, Kristin Pratico, Reid Oakes

**Affiliations:** 1 Strategy and Entrepreneurship, Odette School of Business, University of Windsor, Windsor, ON, Canada; 2 Department of Mathematics and Statistics, University of Windsor, Windsor, ON, Canada; 3 Supply Chain Advancement Network in Health, Odette School of Business, University of Windsor, Windsor, ON, Canada; 4 University of Massachusetts at Lowell, Lowell, MA, United States; 5 HIMSS, Chicago, United States

**Keywords:** digital health, health services, hospitals, medicare, social deprivation, uncompensated care, United States

## Abstract

**Objective:**

The objective of this research is to examine the relationship between digital maturity and hospital care provided to patients who are living with high social disparities.

**Materials and methods:**

This cross-sectional observational study used digital maturity data from 2,247 acute-care hospitals in the United States, linked to CMS Cost Report data, CMS Hospital Service Area data, and ZIP-code-level Social Deprivation Index (SDI) data. EMRAM maturity was classified into three categories: Low=Stage 0, Medium=Stages 1–5, and High=Stages 6–7. The primary measure of social disparity was hospital-level weighted average SDI, dichotomized at the top 20%. Hospital-level administrative indicators included Medicaid caseload, charity-care burden, and bad-debt burden. Multinomial logistic regression was used as the primary model, adjusted for hospital characteristics and U.S. region. Results are reported as average differences in predicted probabilities of Low, Medium, and High EMRAM maturity. Pearson correlations and principal component analysis were used to assess convergence between SDI and hospital-level administrative proxy indicators.

**Results:**

Hospitals treating patient populations in communities with the top 20% of SDI were more likely to have Low EMRAM maturity and less likely to have High EMRAM maturity. In the adjusted multinomial logistic regression model, hospitals in the top SDI category were 5.9 percentage points more likely to have Low EMRAM maturity and 4.6 percentage points less likely to have High EMRAM maturity; the association with Medium EMRAM maturity was not statistically significant. Medicaid caseload and bad-debt burden were not significantly associated with EMRAM maturity. Charity-care burden was associated with EMRAM maturity differently by hospital ownership. Among nonprofit hospitals, top charity-care burden was associated with lower probability of Low EMRAM maturity and higher probabilities of Medium and High EMRAM maturity. Among proprietary hospitals, top charity-care burden was associated with lower probability of Low EMRAM maturity and higher probability of Medium EMRAM maturity, but not High EMRAM maturity. Among government hospitals, charity-care burden was not significantly associated with EMRAM maturity.

**Discussion and conclusions:**

Hospitals serving populations with the highest social disparities were more likely to have Low EMRAM maturity and less likely to have High EMRAM maturity, suggesting that patients in these communities may have reduced access to hospitals with advanced digital maturity that supports coordinated, data-driven, safe, and high-quality care. Hospital administrative measures, including Medicaid caseload, uncompensated care, charity care, and bad debt, did not measure the same construct as community-level social deprivation. Charity-care burden was not uniformly associated with digital maturity across all hospitals; rather, its association differed by ownership type, with the strongest pattern observed among nonprofit hospitals. These findings should be interpreted as observational associations from pre-pandemic data and not as evidence of causal relationships.

## Background and significance

Populations experiencing high social disparities are more likely to receive care from multiple and diverse healthcare facilities (1) and experience repeated visits to emergency departments (ED) rather than access to regular healthcare provider (2, 3). The use of multiple care settings results in data silos, whereby data is stored in multiple care organizations, which limits access to data for both patients and their providers when digital maturity is underdeveloped. Digital maturity is the extent to which digital systems are leveraged for high quality healthcare (4). Digital maturity limitations pose challenges for populations experiencing high social disparities as with limited access to digital tools (such as electronic medical records) they have no access to their personal health data and every health decision relies on either the patient's ability to recall data or health information, or the clinician's ability to piece together patient information to inform care decisions. Further, populations facing social disparities are less likely to have a readily accessible usual source of care (5), such as a family doctor, and may instead rely on fragmented sources like emergency departments across digitally unconnected organizations. Everson et al. (2017) found that hospitals with lower interoperability were more likely to serve marginalized populations, limiting clinicians’ ability to access patient data across settings and increasing the risk of errors and care delays. In a follow-up study, Everson et al. (2023) demonstrated that these interoperability gaps were associated with lower quality of emergency department care for these populations, as clinicians lacked timely access to external records. These findings support the argument that digital maturity is a key structural factor influencing equity in care quality and access. These populations have a higher prevalence of complex chronic health conditions associated with poorer health outcomes (6) and continue to have poorer health outcomes despite technological improvements (7).

The prevalence of chronic health conditions disproportionately affects populations experiencing social disparities (8, 9). Populations experiencing social disparities have been described in terms of socioeconomic factors such as income level, social capital, ability to access healthcare services (10), and limitations in access to care due to economic, geographic (e.g., rural vs. urban), and social factors such as education and transportation (11, 12). In this study, we use the term social disparities as described in the American Community Survey (ACS), which refers to area-level socioeconomic disadvantage, reflecting outcomes of systemic inequities such as poverty, limited education, and reduced access to services. While the term social disparities is similar to social determinants of health, it emphasizes measured disparities in lived conditions across populations (13).

Digital health transformation has been associated with more equitable and sustainable healthcare and improved population health outcomes (14–17). Transformation towards digitally advanced healthcare systems requires not only the adoption of digital tools and technologies, but the integration of these technologies into workflows to re-design and optimize care delivery strategies that strengthen performance outcomes (18, 19). Digital transformation has been associated specifically with reduced costs, improved patient experience, greater workforce satisfaction, and advanced quality and safety outcomes (20–23). In the U.S., national policy efforts such as the HITECH Act of 2009 laid the foundation for EMR adoption by providing financial incentives, though they did not fully address the advancement of interoperability or digital maturity across hospitals. Progress towards digital health transformation is assessed using digital maturity measures (24), which quantify and measure adoption and use of digital technologies to securely capture, store, and flow data seamlessly to the point of care. This provides evidence to inform decisions and to advance strategic priorities of the organization.

A foundational dimension of digital maturity is interoperability, which is defined as the exchange of health data and the seamless and automated sharing of data between clinician teams, patients, and organizations within and across health systems (18). A critical feature of interoperability is the capacity to share and exchange data from varied sources such as patient-reported data, clinical notes, specialist consults, laboratory tests, pharmacy orders, and cardiology and diagnostic imaging reports (25). The interoperable flow of patient data from multiple and diverse sources enables clinicians to access patient data readily to inform data-driven decisions to diagnose, triage, refer, and manage patient care (26). For example, a patient discharged from the hospital with a new diagnosis of heart failure may be seen by their primary care physician within days; if the clinician can access the discharge summary, medication list, and test results electronically, they can adjust the treatment plan and avoid duplicate testing or conflicting prescriptions. The interoperability of data support patients in obtaining the information to inform their care decisions (27) and provides access to digital services, such as virtual care, telehealth, and robust communication between patients and clinicians (28). Interoperability has been associated with improved patient outcomes (such as reduced rates of infections and improved communication with providers) (29), improved access to care (20, 30), and greater access to services like telehealth or virtual care (20, 21).

Digital maturity strengthens interoperability to enable patients to have greater control and autonomy over their health and care, provides access to their health data to monitor progress towards health and wellness goals and inform health decisions, and improves health literacy (31) to achieve desired health outcomes (3, 32, 33). Evidence of outcomes associated with advanced digital maturity, and interoperability in particular, includes reduced adverse events such as medication errors, hospital-acquired infections, diagnostic delays, and duplicate testing, each of which can compromise patient safety and increase healthcare costs (28, 34, 35). As health systems continue to advance digital transformation efforts to strengthen health system performance, evidence is needed of digital maturity and interoperability’s influence on patient access to care and health outcomes of unique populations, such as those experiencing high social disparities, to inform leadership strategies in advancing equity of access to care and achieving equitable health outcomes for these populations (2, 3).

While digital maturity has been linked to both advances in health system performance and population and equity outcomes (3), significant variation exists to the degree in which health organizations have advanced digital maturity and interoperability to support integrated care delivery, particularly for populations with highly complex health conditions (3). Digital maturity requires advanced interoperability, which has been found to be less well developed in smaller, more rural, independent hospitals compared to larger, urban hospital systems (36–38). A recent study revealed that a sample of 74% U.S. hospitals scored Stage 0 (out of 7) on the digital maturity assessment using the Electronic Medical Record Adoption Model (EMRAM) (39). EMRAM is a digital maturity assessment tool designed to measure healthcare organization interoperability on a scale of 0 to 7 (39). The indicator requirements for hospitals to meet EMRAM Stage 1 maturity include integration of lab, pharmacy, diagnostic imaging, and cardiology information systems, and resilience management systems in the event of a disruption (39). Hospitals achieving EMRAM Stage 6 or 7 maturity receive further assessment and validation onsite by a team from the Healthcare Information and Management Systems Society (HIMSS) using advanced indicator requirements, which include patient experience, data exchange, adoption of digital technologies, resilience of information systems, and governance (39). Given that most hospitals do not have mature interoperable exchange of patient data, populations experiencing high social disparities may be at greater risk for fragmented, poorly coordinated care due to data silos within and across care settings (3). Everson et al. (2023) found that hospitals serving marginalized communities were significantly less likely to have robust interoperability capabilities, limiting clinicians’ access to complete patient information. This gap increases the likelihood of care delays, duplicated services, and medical errors—especially for patients who receive care across multiple, disconnected facilities. The seamless flow of data between laboratory, diagnostic imaging, pharmacy, cardiology, and electronic medical records (EMRs) for each patient within and across care settings can help reduce harm and poor patient outcomes of care transitions across settings, which are common occurrences for populations experiencing high social disparities (3).

The purpose of this research was to empirically examine digital maturity in U.S. hospitals that provide care for populations with high social disparities and to assess how digital maturity is associated with area-level social deprivation, hospital-level administrative indicators of service burden, and hospital characteristics. We hypothesize that hospitals serving populations with high social disparities may have less capacity for interoperable exchange of data due to limited digital maturity advancement, resulting in reduced ability to support the flow of data to patients and clinicians across multiple settings to inform care decisions. Examining differences in hospital characteristics, digital maturity, and social disparities among patient populations may provide evidence to guide leadership strategies and policy initiatives aimed at strengthening digital maturity and ensuring that hospitals have the digital capacity to offer quality and safe care to populations experiencing high social disparities.

### Conceptual framework

**Figure 1:**
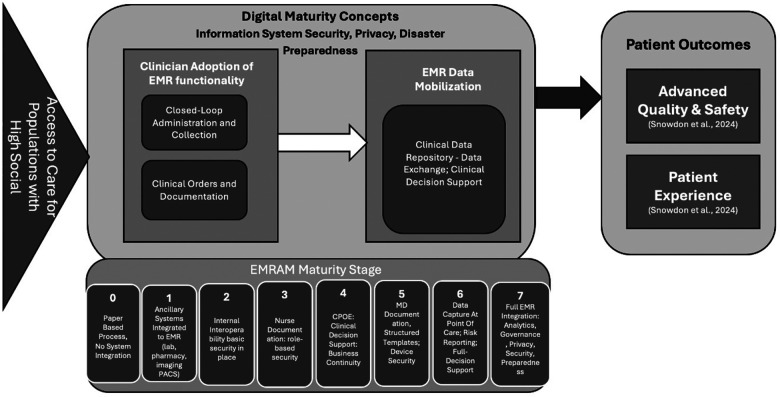
Conceptual framework.

Figure 1 illustrates the conceptual framework of this study. The study's digital maturity was measured using EMRAM, which gauges four concepts: clinician adoption and use of EMR technologies to capture data and document care delivery; information system security, privacy, and resilience to disruptions; and the mobilization of data to support clinical decision-making and decision support. Digital maturity measured by EMRAM progresses across seven stages of maturity, with key milestones illustrated for each stage (Figure 1). Advanced digital maturity has been associated with patient outcomes such as greater quality and safety as well as improved patient experience (40). The focus of this study was to examine whether these patient outcomes of digital maturity are accessible to populations with high levels of social disparities in U.S. hospitals. As mentioned earlier, hospitals scoring at a 6 or 7 digital maturity stage were further assessed onsite by a HIMSS team to validate their advanced digital maturity stage. An EMRAM Stage 0 assessment determines that data from laboratory, pharmacy, imaging, and interventional cardiology have not been integrated into the EMR, resulting in significant data silos that preclude interoperable data flow to the point of care. For example, to achieve Stage 1 maturity for EMRAM, hospitals must have fully integrated laboratory, imaging, pharmacy, and cardiology information systems into the EMR with data such as diagnostic imaging reports, lab results, and medication profiles automatically flowing into the EMR. For the purposes of this study, hospitals at EMRAM Stage 0 were classified as having underdeveloped digital maturity, which may indicate that the organization relies on paper-based documentation and data capture and have not started their digital transformation journey. EMRAM Stage 0 may also indicate that the hospital organization has implemented an EMR, but it has not integrated or connected other information systems (e.g., lab, pharmacy, imaging) into the EMR system, which limits it to basic EMR functionality.

Figure 1 Conceptual Framework of Digital Maturity and Access to Care for Populations with High Social Disparities.

## Method

This study used a cross-sectional design and gathered data from multiple sources to examine the association between digital maturity and access to quality and safe care by populations experiencing high levels of social disparities seeking care at U.S. hospitals.

### Data sources

Four data sources were leveraged for this analysis: CMS Cost Report data, Social Deprivation Index data, CMS Hospital Service Area data, and EMRAM data to measure digital maturity. The first data accessed for this study was the CMS *Healthcare Cost Report Information System of 2018* (41). From this data, we extracted the following variables:

- Medicaid Case Load (MCL): a proportion of total discharges that come from Medicaid patients.
- Burden of Uncompensated Care (UCC): a measure of hospital care provided for which no payment was received from the patient or insurer. The UCC is defined as the sum of two components: (i) proportion of charity care cost (CC) out of the hospital's total operating expenses and (ii) proportion of bad debt (BD) out of the hospital's total operating expenses. BD consists of services for which hospitals anticipated but did not receive payment (42). BD is defined by CMS as unpaid Medicare deductibles and coinsurance amounts that providers cannot collect from patients, which are thus treated as bad debts and factored into how providers are reimbursed by Medicare (43).

The UCC, its two components, and MCL were used as hospital-level administrative indicators that may reflect aspects of service burden among hospitals caring for populations with high social disparities. These measures were not treated as direct measures of social deprivation, but rather as proxy indicators that may capture related dimensions such as payer mix, uncompensated care, charity-care provision, bad-debt burden, and financial exposure. Hospital characteristics data accessed in the CMS data files included data on teaching status, urban or rural location, type of ownership (e.g., government, non-profit, for-profit), hospital size (e.g., small=0–149 beds; medium=150–399 beds; large=400 + beds), and critical-access hospital designation.

The second data set consisted of SDI, reported by ZIP code, based on census data from 2014 to 2018. This data was retrieved from the Robert Graham Center (44), a research and policy organization affiliated with the American Academy of Family Physicians with the mandate of improving population health and primary care (44). The SDI is a composite measure of area-level deprivation based on seven demographic characteristics collected as part of the American Community Survey. We used ZIP-code level SDI scores as provided by the Robert Graham Center. These scores are calculated using principal component analysis of the seven standardized variables associated with social disparities, resulting in a continuous index ranging from 0 to 100, with higher scores indicating greater social deprivation. The seven characteristics measured to determine social disparities include the percentage of: people living in poverty, people with less than 12 years of education, single-parent households, people living in rented housing units, people living in an overcrowded housing unit, households without a car, and non-employed adults under 65 years of age (13). The seven components of the SDI are comparable to social determinants of health defined by the World Health Organization (WHO) (45). However, the SDI does not measure social inclusion, discrimination, or access to affordable and quality health services, which are included in the social determinants of health defined by the WHO. This research examined access to health services at hospitals with advanced or underdeveloped digital maturity for the U.S. population experiencing the top 20% on the SDI.

The third data set was the volume of patients from each ZIP code served by a given hospital. This data was retrieved from CMS's Hospital Service Area File of 2018 (46). Building on previous work (3), the SDI for each ZIP code and the volume of patients served by each hospital were combined to compute a weighted average SDI for each hospital. The weights were defined as the proportion of the hospital's patients coming from a particular ZIP code divided by the total of such proportions for all ZIP codes served by that hospital. A higher weighted average SDI score means that the hospital serves patients with higher social disparities.

The fourth data set included in this analysis was data from hospitals that had completed digital maturity assessments using EMRAM for 2018 and 2019 (24). EMRAM data for this study was limited to data collected in 2018 and 2019 in order to align this data to the same year as the SDI data and CMS’s Hospital Service Area File of 2018, where the SDI scores for each hospital had already been linked and reported in the CMS database for this time period.

HIMSS made the EMRAM data available for this analysis. EMRAM is an eight-stage framework developed by HIMSS to assess the extent to which hospitals have adopted and integrated the use of EMR technologies integrated with software systems to enable interoperability across the organization. Hospitals are scored from Stage 0 (no integration of EMR with pharmacy, lab, or imaging information systems) to Stage 7 (fully integrated information systems with robust interoperability and flow of data across the organization). EMRAM provides a standardized, internationally used measure of digital maturity and is widely applied in health policy, infrastructure planning, and system-level benchmarking. The model has been widely adopted with 5,501 U.S. hospitals and 10,389 hospitals globally having assessed their digital maturity since its release in 2018 (41). A number of studies have examined and validated the EMRAM (39, 44, 47–49). In 2018, it was made available to hospitals online, at no cost, to provide hospitals with a measurement of their digital maturity to inform strategies to advance digital transformation. Hospital teams responded online to EMRAM's 167 survey items using a 5-Point Likert Scale ranging from not enabled to fully enabled. An embedded algorithm then generated a digital maturity stage on a 7-point scale ranging from 0 to 7.

Hospitals attaining Stage 6 or 7 maturity have successfully achieved milestones such as ≥95% staff adoption and deployed advanced EMR functions, including computerized physician order entry (CPOE), bar code medication administration (BCMA), integration of data from multiple internal and external sources into clinical settings, alerts to identify risk of errors, and automated digital tools to track progress of patient outcomes. These EMR functions are designed to support clinician teams to deliver quality and safe care, with reduced errors related to physician orders or medication administration. Figure 1 illustrates each stage of digital maturity. For this analysis, EMRAM maturity was classified into three categories: Low=Stage 0, Medium=Stages 1–5, and High=Stages 6–7. Because relatively few hospitals were observed at each individual stage from 1 through 5, these stages were combined into a single Medium digital maturity category rather than analyzed separately. Hospitals that scored Stage 0 may wait to retake the assessment until they have made significant progress, which may serve as motivation to profile their accomplishments when they reach Stage 6 or 7. This study was based exclusively on de-identified, publicly available data sources and did not require Institutional Review Board (IRB) approval.

The initial EMRAM dataset included 3,355 hospitals with available EMRAM outcome data. Among these hospitals, 2,247 had complete data for all analytic variables and were included in the final complete-case analytic sample, while 1,108 hospitals (33.0%) were missing at least one linked analytic variable. Missingness was most common for Medicaid caseload category (*n* = 817; 24.4%), uncompensated-care burden category (*n* = 760; 22.7%), SDI and SDI-top classification (*n* = 474 each; 14.1%), teaching status, urban/rural location, and critical-access status (*n* = 341 each; 10.2%), ownership (*n* = 321; 9.6%), and region (*n* = 1; 0.03%). EMRAM maturity and bed-size category were complete for all 3,355 hospitals. Because the primary regression analyses required complete covariate information, hospitals with missing linked analytic variables were excluded from the complete-case analyses.

Missingness arose from several linkage and reporting issues across the EMRAM, CMS Cost Report, and CMS Hospital Service Area data sources. Some EMRAM hospitals could not be matched to a CMS Certification Number, which may reflect name changes, multiple facility names, inactive or consolidated CMS identifiers, or facilities that were not Medicare-certified institutional providers required to submit annual cost reports. Other hospitals had matched CMS identifiers but were missing demographic or cost-report variables. Missingness in Medicaid caseload and uncompensated-care measures likely reflected incomplete reporting of the underlying CMS cost-report fields. Missing SDI values resulted from missing or insufficient CMS Hospital Service Area data, including suppression of ZIP-code-level counts when cell sizes were fewer than 11 patients to protect confidentiality. One additional hospital was excluded because it was located in Puerto Rico and therefore was not assigned to a U.S. Census region.

To further assess generalizability, we compared the 2,247 hospitals in the final EMRAM analytic sample with hospitals excluded because of incomplete linked data and with the remaining hospitals in the CMS-linked hospital file that were not included in the analytic sample. These comparisons were used to evaluate whether hospitals included in the final analytic sample differed from excluded EMRAM hospitals or from the broader CMS-linked hospital population with respect to EMRAM maturity and hospital characteristics.

### Statistical analysis

The primary outcome in this study was EMRAM maturity classified into three ordinal categories (0 = Low, 1–5 = Medium, and 6, 7 = High). Due to the small sample size of hospitals at maturity stages 1–5, we elected to combine these categories.

Marginalization measures, namely, SDI, MCL, CC, and BD, were the main predictor variables, and hospital characteristics were used as covariates. Although the SDI is a continuous variable, we followed established methods in the literature (2, 3), and dichotomized the SDI at the top quintile (e.g., top 20%) in order to support meaningful comparison of the results of this study with current evidence. This method enables easier interpretation and supports policy-relevant comparisons, particularly in identifying hospitals that serve the most socially disadvantaged populations. Hospitals whose SDI scores were in the top 20% were classified as “top,” indicating that they serve patients living in ZIP codes with the highest level of social disparities, and hospitals whose patients live in ZIP codes with SDI scores in the lowest 80% were classified as “bottom.” Similar categorization into “top” and “bottom” were applied to MCL, UCC, CC, and BD measures.

SDI, MCL, UCC, CC, and BD capture related, but conceptually distinct, dimensions of social disadvantage and hospital service burden. Therefore, we conducted a planned validity assessment before interpreting these measures in the multivariable models. The SDI was treated *a priori* as the primary measure of area-level social deprivation because it is derived from ZIP-code-level socioeconomic characteristics. In contrast, MCL, UCC, CC, and BD were treated as hospital-level administrative proxy measures that may reflect aspects of the populations served and the financial burden of care, but are not direct measures of social deprivation. We expected these measures to show some convergence with SDI, but not complete overlap. To assess the degree of empirical convergence among these measures, we examined Pearson correlations among SDI, MCL, UCC, CC, and BD and used principal component analysis (PCA) to evaluate whether they loaded onto common underlying dimensions. This analysis was intended to clarify whether the administrative proxy measures could reasonably be interpreted alongside SDI or whether they captured distinct phenomena, such as payer mix, uncompensated care burden, charity-care provision, or bad-debt burden.

Because EMRAM maturity was classified into three ordered categories, an ordinal logistic regression model was initially examined. However, post-estimation tests of the proportional odds assumption indicated that this assumption was not satisfied. The Score test of the parallel regression assumption was statistically significant, *χ*2(16) = 119.90, *p* < 0.001, and the Brant test was also statistically significant, *χ*^2^(16) = 60.50, *p* < 0.001. Therefore, multinomial logistic regression was used for the primary multivariable analysis because it does not require the proportional odds assumption and allows associations to differ across Low, Medium, and High EMRAM maturity categories. Low EMRAM maturity was specified as the reference outcome category.

We used SDI, MCL, CC, and BD as predictors while controlling for hospital characteristics, including bed size, urban/rural location, teaching status, critical-access status, ownership, and U.S. region.

We evaluated candidate interaction terms between the main predictors and hospital demographic covariates and used Akaike Information Criterion (AIC) to guide model selection. The final model retained the interaction between charity care and ownership, indicating that the association between charity-care burden and EMRAM maturity differed by ownership type.

In the multivariate analyses, instead of the burden of cost of uncompensated care, we opted to use its constituent data points directly, namely the burdens of BD and CC. This analysis was designed to better understand the drivers of any relationship between digital maturity and UCC. The results of the multinomial logistic regression model are presented as average marginal effects (AMEs) of the predictors of the probabilities of hospitals being in each EMRAM maturity category: Low, Medium, and High.

Model diagnostics were conducted to assess the appropriateness of the multinomial logistic regression model. An alpha level of 0.05 (5%) was used to determine significance in all statistical tests in this study. Regression analyses were completed using Stata; Pearson correlations and PCA were completed using R (50).

## Results

### Hospital sample characteristics

Table 1 summarizes the characteristics of hospitals included in this study sample (*n* = 2,247). Of these hospitals, 1,978 (88.0%) scored at EMRAM Stage 0, 73 (3.2%) scored at Stages 1 through 5, and 196 (8.7%) were highly digitally mature, validated at EMRAM maturity Stage 6 or 7. The majority of hospitals were nonprofit (*n* = 1,225; 54.5%), followed by government-owned hospitals (*n* = 571; 25.4%) and proprietary hospitals (*n* = 451; 20.1%). Eight hundred seven hospitals (35.9%) were critical-access hospitals, and 448 (19.9%) were teaching hospitals. The study sample included 1,546 small hospitals with 0–149 beds (68.8%), 527 medium-sized hospitals with 150–399 beds (23.5%), and 174 large hospitals with 400 or more beds (7.7%). Regionally, 663 hospitals were located in the Midwest (29.5%), 271 in the Northeast (12.1%), 923 in the South (41.1%), and 390 in the West (17.4%).

**Table 1:** Summary of hospital characteristics used in analysis.

Variable	*N* (%)
EMRAM level achieved	
Low (0)	1,978 (88.0%)
Medium (1–5)	73 (3.2%)
High (6–7)	196 (8.7%)
Teaching status	
Non-Teaching	1,799 (80.1%)
Teaching	448 (19.9%)
Urban/Rural location	
Rural	1,325 (59.0%)
Urban	922 (41.0%)
Ownership	
Governmental	571 (25.4%)
Nonprofit	1,225 (54.5%)
Proprietary	451 (20.1%)
Bed size	
0–149	1,546 (68.8%)
150–399	527 (23.5%)
400+	174 (7.7%)
Critical access status	
Critical Access	807 (35.9%)
Not Critical Access	1,440 (64.1%)
Region	
Midwest	663 (29.5%)
Northeast	271 (12.1%)
South	923 (41.1%)
West	390 (17.4%)
Total	2,247 (100.0%)

Table 1 describes the characteristics of the 2,247 hospitals included in the analysis, including EMRAM level, teaching status, urban/rural location, ownership, bed size, critical-access status, and U.S. region.

Compared with the remaining CMS-linked hospitals, hospitals in the final EMRAM analytic sample were more likely to be small, rural, critical-access, nonprofit, and governmental hospitals, and less likely to be large, urban, teaching, or proprietary hospitals. Specifically, small hospitals represented 76.3% of the final EMRAM analytic sample compared with 68.5% of the remaining CMS-linked hospitals, while large hospitals represented 4.4% compared with 8.1%. Critical-access hospitals were also more common in the final EMRAM analytic sample (36.2% vs. 14.6%), as were rural hospitals (59.3% vs. 29.6%). Nonprofit hospitals represented 54.4% of the final EMRAM analytic sample compared with 46.8% of the remaining CMS-linked hospitals, while proprietary hospitals were less common (20.1% vs. 35.6%). Teaching hospitals were also less common in the final EMRAM analytic sample than in the remaining CMS-linked hospitals (19.6% vs. 25.1%). These differences indicate that the final EMRAM analytic sample may not fully represent the broader CMS-linked hospital population.

### Analyses of social disparities

In this study, the SDI was considered as the more accurate measure of social disparities experienced by patients, when compared to proxy measures of social disparities that are embedded in hospital-level data. We examined the strength of the relationship between these proxy indicators (MCL, UCC, CC, BD) and the SDI. Accordingly, Table 2 reports the percentages of hospitals that fall within the top 20% of each proxy measure (MCL, UCC, BD, CC), and at the same time in the top 20% with respect to the SDI. Four hundred twenty-nine hospitals served populations in the top 20% of average SDI, and of those, approximately 30% fall in the top 20% of MCL, UCC, and BD. An exception is the charity care burden, which has only 25.9% of the 429 hospitals in its top 20%. This analysis revealed that although UCC is somewhat concordant with the SDI, its constituent variables (i.e., cost of bad debt and cost of charity care) are not equally concordant with the SDI, whereby UCC is more heavily influenced by BD. In other words, hospitals that have higher levels of charity care costs are not necessarily serving populations living in ZIP codes with higher social disparities.

**Table 2:** SDI classification by hospital-level administrative proxy indicators.

Proxy measures	SDI	
	Bottom (*N* = 1818)	Top (*N* = 429)
Medicaid Case Load		
Bottom	1489 (81.9%)	299 (69.7%)
Top	329 (18.1%)	130 (30.3%)
Uncompensated care		
Bottom	1513 (83.2%)	297 (69.2%)
Top	305 (16.8%)	132 (30.8%)
Bad Debt		
Bottom	1507 (82.9%)	298 (69.5%)
Top	311 (17.1%)	131 (30.5%)
Charity Care		
Bottom	1467 (80.7%)	318 (74.1%)
Top	351 (19.3%)	111 (25.9%)

SDI, Social Deprivation Index.

Values are frequency (column percentage). SDI top=hospitals serving populations in the highest 20% of average SDI scores; SDI bottom=hospitals serving populations in the lowest 80% of average SDI scores. For each administrative proxy indicator, Top=highest 20% and Bottom=lowest 80%.

Table 2 reports the distribution of hospitals across top and bottom categories of Medicaid caseload, uncompensated care, bad debt, and charity care, stratified by whether hospitals served populations in the top 20% or bottom 80% of SDI. Among hospitals serving populations in the top SDI group, about 30% were also in the top 20% of Medicaid caseload, uncompensated care, and bad debt, while 25.9% were in the top 20% of charity care.

**Figure 2:**
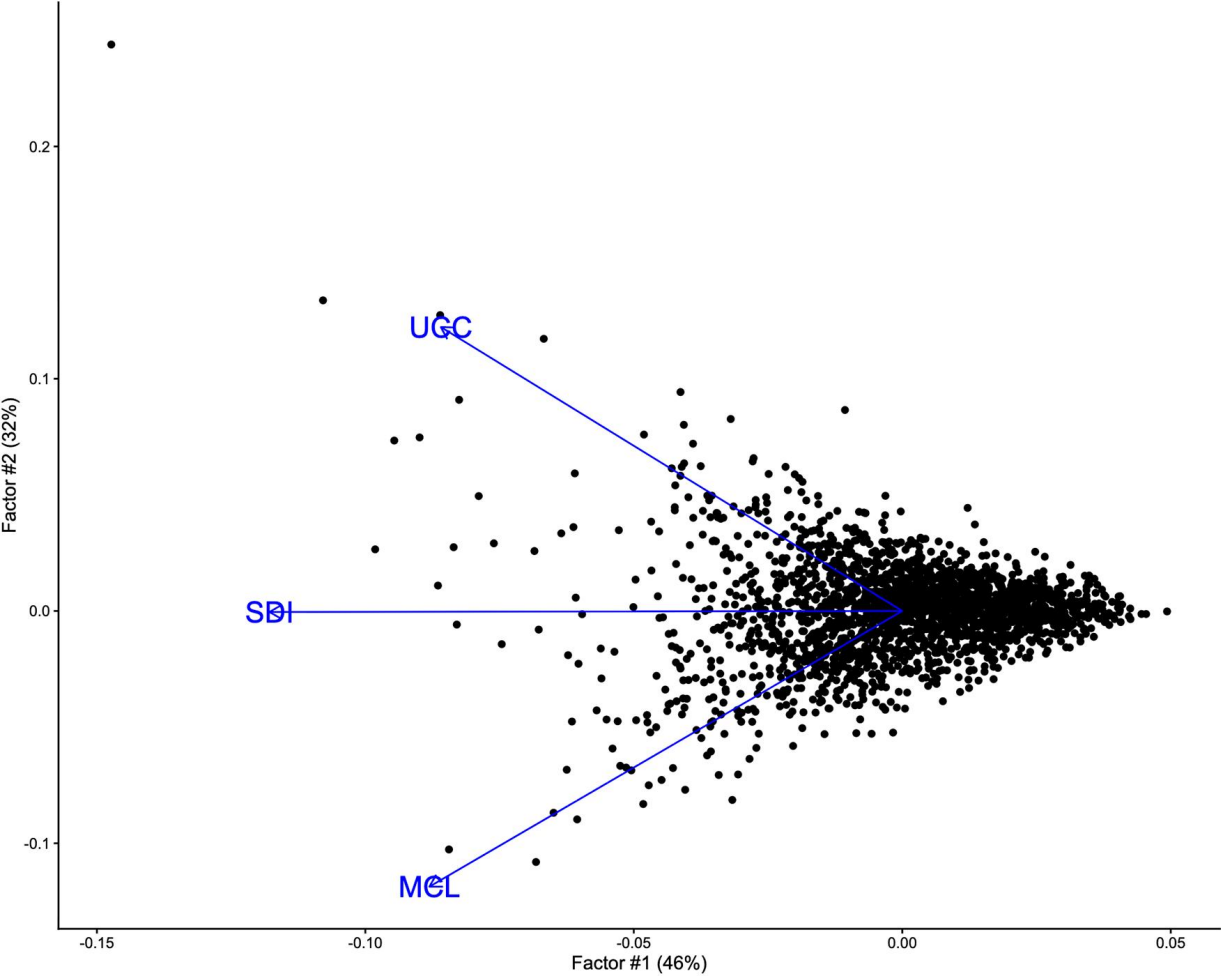
Correlations and distributions of SDI and hospital-level administrative proxy indicators.

We analyzed the correlations among these proxy measures and the SDI (Figure 2). We found that the correlations among the measures were statistically significant but not very high. Pearson correlations between the SDI and each of MCL and UCC were 0.22 (95% CI: 0.213–0.290), and 0.23 (95% CI: 0.205–0.282), respectively.

**Figure 3:**
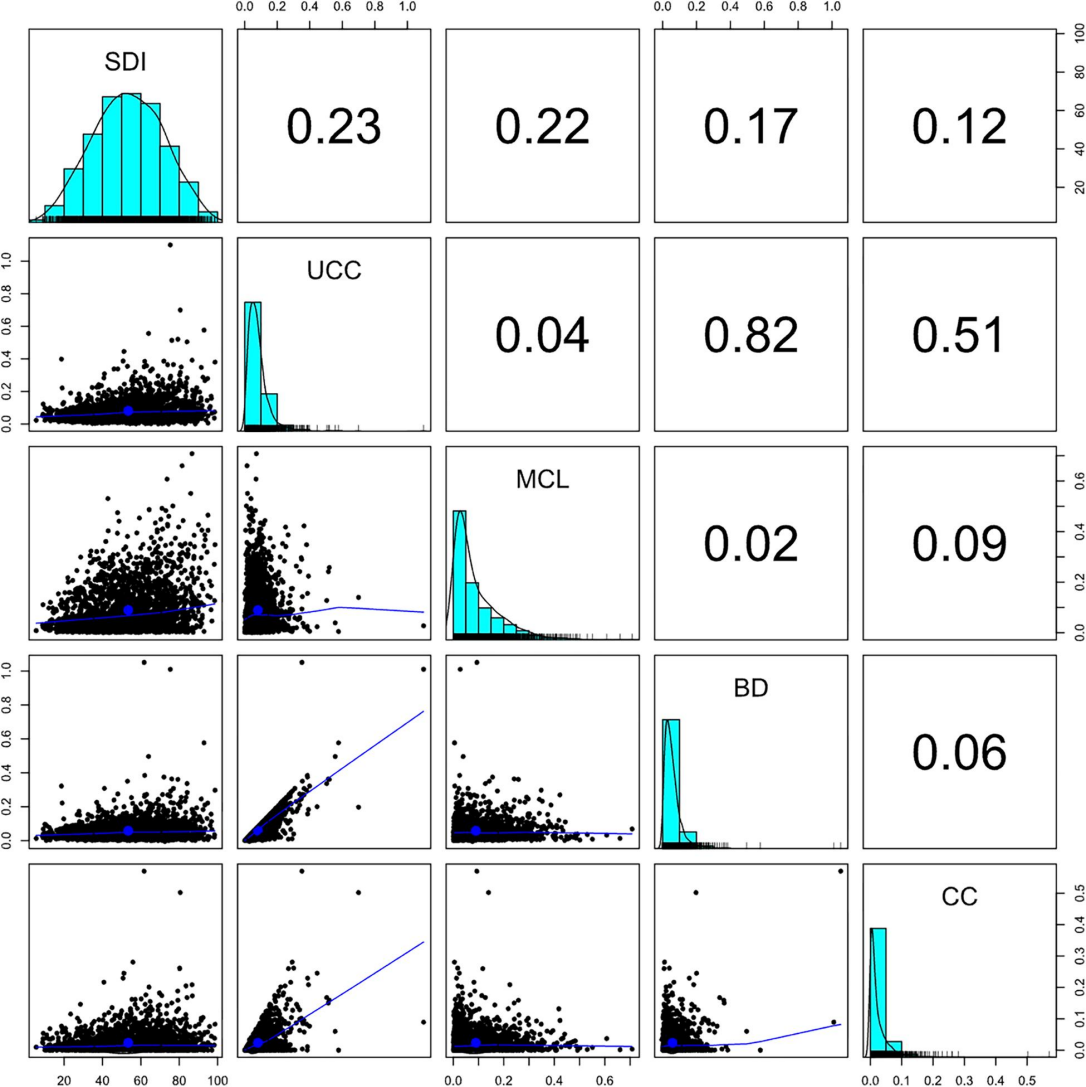
Principal component analysis of SDI and hospital-level administrative proxy indicators. Correlations among all measures of marginalization and their distributions(upper Panel). Principal Component Analysis (PCA) showing alignment of the three measures of marginalization with the main two underlying principal factors(lower panel).

Further, we performed PCA to explore whether SDI and the hospital-level administrative indicators loaded onto common underlying dimensions (Figure 3). The first two principal components explained 78% of the information in these measures. The PCA suggested that SDI and the hospital-level administrative indicators capture related but distinct dimensions. SDI appeared most closely aligned with area-level social deprivation, while the administrative indicators reflected other aspects of hospital costs, payer mix, uncompensated-care burden, and patient population characteristics.

### Digital maturity and social disparity

This analysis used the two constituent variables of UCC, namely BD and CC, when modeling the association between digital maturity and measures of social disparity. These two variables contribute different levels of influence on the UCC composite measure and were examined separately to better understand their associations with EMRAM maturity. Table 3 reports the results of the adjusted multinomial logistic regression analysis, which indicate that hospitals treating patients experiencing the highest social disparities, defined as populations living in ZIP codes with the top 20% average SDI scores, were significantly more likely to have low digital maturity and significantly less likely to have high digital maturity. Specifically, hospitals in the top SDI category were 5.9 percentage points more likely to have low digital maturity (AME: 0.059, 95% CI: 0.029, 0.089), and 4.6 percentage points less likely to have high digital maturity (AME: −0.046, 95% CI: −0.071, −0.021). The association with medium digital maturity was not statistically significant after region adjustment (AME: −0.013, 95% CI: −0.031, 0.004).

**Table 3:** Adjusted average differences in predicted probabilities of high, Medium, and Low EMRAM maturity from the region-adjusted multinomial logistic regression model.

Independent variable and comparison	High	Medium	Low
SDI Top vs. Bottom	−0.046** (−0.071, −0.021)	−0.013 (−0.031, 0.004)	0.059** (0.029, 0.089)
Burden of Charity Care Top vs. Bottom, among Government hospitals	0.047 (−0.014, 0.108)	−0.022 (−0.050, 0.007)	−0.025 (−0.092, 0.041)
Burden of Charity Care Top vs. Bottom, among Nonprofit hospitals	0.079** (0.026, 0.132)	0.045* (0.009, 0.081)	−0.124** (−0.185, −0.063)
Burden of Charity Care Top vs. Bottom, among Proprietary hospitals	0.005 (−0.015, 0.026)	0.077* (0.014, 0.140)	−0.083* (−0.148, −0.017)
Burden of Bad Debt Top vs. Bottom	<0.001 (−0.031, 0.031)	0.004 (−0.015, 0.023)	−0.004 (−0.040, 0.032)
Medicaid Caseload Top vs. Bottom	0.001 (−0.029, 0.030)	0.006 (−0.017, 0.028)	−0.007 (−0.042, 0.029)
Critical Access Status Non-Critical Access vs. Critical Access	0.007 (−0.028, 0.042)	0.006 (−0.013, 0.026)	−0.013 (−0.053, 0.026)
Teaching Status Teaching vs. Non-Teaching	0.023 (−0.010, 0.056)	−0.002 (−0.029, 0.025)	−0.021 (−0.062, 0.020)
Urban/Rural Location Urban vs. Rural	0.056** (0.027, 0.085)	−0.009 (−0.027, 0.010)	−0.047** (−0.081, −0.014)
Bed Size Medium vs. Small	0.029 (−0.003, 0.061)	−0.010 (−0.032, 0.011)	−0.019 (−0.056, 0.019)
Bed Size Large vs. Small	0.080** (0.023, 0.136)	−0.017 (−0.045, 0.011)	−0.063* (−0.124, −0.001)
Region Northeast vs. Midwest	0.004 (−0.028, 0.036)	−0.039** (−0.063, −0.015)	0.035 (−0.005, 0.074)
Region South vs. Midwest	0.029 (−0.001, 0.059)	−0.027* (−0.050, −0.004)	−0.002 (−0.039, 0.035)
Region West vs. Midwest	0.012 (−0.024, 0.049)	−0.032** (−0.056, −0.008)	0.019 (−0.022, 0.061)
Ownership Nonprofit vs. Government, among bottom Charity Care hospitals	0.066** (0.034, 0.098)	−0.006 (−0.025, 0.013)	−0.060** (−0.097, −0.024)
Ownership Nonprofit vs. Government, among top Charity Care hospitals	0.098** (0.026, 0.170)	0.061** (0.020, 0.102)	−0.159** (−0.239, −0.079)
Ownership Proprietary vs. Government, among bottom Charity Care hospitals	−0.036** (−0.061, −0.011)	−0.027** (−0.046, −0.008)	0.063** (0.032, 0.094)
Ownership Proprietary vs. Government, among top Charity Care hospitals	−0.078* (−0.137, −0.018)	0.072* (0.006, 0.138)	0.006 (−0.082, 0.093)

SDI, Social Deprivation Index; AME, average marginal effect; EMRAM, Electronic Medical Record Adoption Model.

Values are AMEs with 95% confidence intervals from a region-adjusted multinomial logistic regression model. Each AME represents the average difference in the predicted probability of being in the listed EMRAM maturity category for the comparison shown. For each proxy measure, Top=highest 20% and Bottom=lowest 80%. The model includes a charity care×ownership interaction; therefore, charity-care and ownership effects are reported conditionally. The model adjusts for SDI, charity-care burden, bad-debt burden, Medicaid caseload, critical-access status, teaching status, urban/rural location, bed size, ownership, and U.S. region. Midwest was the reference region.

* *p* < 0.05; **p < 0.01.

Table 3 reports adjusted average differences in predicted probabilities of High, Medium, and Low EMRAM maturity for each comparison.

The relationship between digital maturity and burden of CC differed by hospital ownership. Among government hospitals, top charity-care burden was not significantly associated with low, medium, or high digital maturity. Among nonprofit hospitals, top charity-care burden was associated with a 12.4 percentage-point lower probability of low digital maturity (AME: −0.124, 95% CI: −0.185, −0.063), a 4.5 percentage-point higher probability of medium digital maturity (AME: 0.045, 95% CI: 0.009, 0.081), and a 7.9 percentage-point higher probability of high digital maturity (AME: 0.079, 95% CI: 0.026, 0.132). Among proprietary hospitals, top charity-care burden was associated with an 8.3 percentage-point lower probability of low digital maturity (AME: −0.083, 95% CI: −0.148, −0.017) and a 7.7 percentage-point higher probability of medium digital maturity (AME: 0.077, 95% CI: 0.014, 0.140), but it was not significantly associated with high digital maturity (AME: 0.005, 95% CI: −0.015, 0.026).

MCL and BD were not statistically significantly related to digital maturity, meaning hospitals with high and low digital maturity did not differ significantly in their Medicaid caseload or bad-debt burden. A hospital's critical-access or teaching status also had no statistically significant association with the probability of low, medium, or high digital maturity. However, urban location, bed size, ownership, and U.S. region were associated with EMRAM maturity. Urban hospitals were 4.7 percentage points less likely to have low digital maturity (AME: −0.047, 95% CI: −0.081, −0.014) and 5.6 percentage points more likely to have high digital maturity (AME: 0.056, 95% CI: 0.027, 0.085) than rural hospitals. Large hospitals, with 400 or more beds, were 6.3 percentage points less likely to have low digital maturity (AME: −0.063, 95% CI: −0.124, −0.001) and 8.0 percentage points more likely to have high digital maturity (AME: 0.080, 95% CI: 0.023, 0.136) than small hospitals with fewer than 150 beds. Compared with hospitals in the Midwest, hospitals in the Northeast (AME: −0.039, 95% CI: −0.063, −0.015), South (AME: −0.027, 95% CI: −0.050, −0.004), and West (AME: −0.032, 95% CI: −0.056, −0.008) had significantly lower probabilities of medium digital maturity. Region was not significantly associated with low or high digital maturity.

Ownership effects also differed according to charity-care burden. Among hospitals in the bottom charity-care group, nonprofit hospitals were 6.0 percentage points less likely than government hospitals to have low digital maturity (AME: −0.060, 95% CI: −0.097, −0.024) and 6.6 percentage points more likely to have high digital maturity (AME: 0.066, 95% CI: 0.034, 0.098), while the difference in medium digital maturity was not statistically significant. Among hospitals in the top charity-care group, nonprofit hospitals were 15.9 percentage points less likely to have low digital maturity (AME: −0.159, 95% CI: −0.239, −0.079), 6.1 percentage points more likely to have medium digital maturity (AME: 0.061, 95% CI: 0.020, 0.102), and 9.8 percentage points more likely to have high digital maturity (AME: 0.098, 95% CI: 0.026, 0.170) compared with government hospitals. Proprietary hospitals showed a different pattern. Among hospitals in the bottom charity-care group, proprietary hospitals were 6.3 percentage points more likely than government hospitals to have low digital maturity (AME: 0.063, 95% CI: 0.032, 0.094), 2.7 percentage points less likely to have medium digital maturity (AME: −0.027, 95% CI: −0.046, −0.008), and 3.6 percentage points less likely to have high digital maturity (AME: −0.036, 95% CI: −0.061, −0.011). Among hospitals in the top charity-care group, proprietary hospitals were 7.2 percentage points more likely to have medium digital maturity (AME: 0.072, 95% CI: 0.006, 0.138) and 7.8 percentage points less likely to have high digital maturity (AME: −0.078, 95% CI: −0.137, −0.018) than government hospitals, while the difference in low digital maturity was not statistically significant (AME: 0.006, 95% CI: −0.082, 0.093).

## Discussion

Digital maturity, and interoperability in particular, has been documented to achieve significant improvements in hospital performance (23, 39, 51–54) Yet, more than 80% of hospitals in this study were at a maturity stage of 0, indicating profound limitations in the access and flow of data to inform decisions for their clinicians and patients. Moreover, populations with the highest social disparities, defined as the top 20% of the SDI, were significantly more likely to receive care from hospitals with low digital maturity and significantly less likely to receive care from hospitals with high digital maturity, compared with hospitals serving populations with lower levels of social disparities. In the adjusted model, the association between SDI and medium digital maturity was no longer statistically significant, but the primary pattern remained: hospitals serving populations in the top SDI category were more likely to have low EMRAM maturity and less likely to have high EMRAM maturity. Notably, digital maturity of hospitals in this study was not significantly different by critical-access or teaching status, nor did hospitals with high vs. low digital maturity vary significantly in terms of their Medicaid caseloads. This result is somewhat unexpected given prior literature suggesting that critical-access and rural hospitals face significant challenges in advancing digital maturity due to limited infrastructure, capital, and workforce capacity (Vest, 2010; Holmgren, Patel, & Adler-Milstein, 2017). One possible explanation is that critical-access designation alone may not capture the broader organizational context, such as network affiliation, access to shared infrastructure, or recent policy incentives that have enabled progress. Similarly, although Medicaid caseload is frequently associated with high service burden and financial constraints, our findings indicate that it may not be a consistent predictor of digital maturity. Further research is needed to explore whether leadership priorities, access to capital investment, regional context, or policy alignment more accurately account for digital transformation progress among these types of hospitals. Thus, these findings should not be interpreted as evidence that resources are unimportant for digital maturity; rather, they suggest that critical-access designation and Medicaid caseload alone may not adequately capture the broader resource environment reflected by hospital size, ownership, urban/rural context, access to capital, shared infrastructure, and regional policy conditions.

The digital maturity limitations among U.S. hospitals in this study may be associated with the “digital divide” experienced by patient populations with the highest levels of social disparities, who are well documented to have higher prevalence of complex health conditions and to rely more heavily on hospitals for health services. Digital maturity at Stage 0 means that neither patients nor their clinician teams at these organizations have sufficient access to health data, such as medication history, laboratory results, and imaging reports, to inform care decisions. The digital divide these patients face may be associated with reduced access to data-driven, coordinated care, which has been linked to lower quality and safety outcomes in hospitals with limited EMR adoption and interoperability capacity (2, 34). Hospitals that have achieved digital maturity advances have significantly stronger quality and safety outcomes (39) and better patient experience (40). Yet, these findings raise the critical question of whether differences in digital maturity across hospitals are associated with imbalances in access to data-driven care and equitable outcomes for populations with high levels of social disparities, particularly given that low digital maturity in this study was not associated with higher Medicaid caseloads or critical-access status.

The study's results, based on the use of EMRAM data from 2018 to 2019, have limitations for understanding U.S. hospitals in 2025, given the substantive changes in digital technologies and health services since the pandemic. However, these findings may offer valuable insights into the shortcomings of digital maturity among U.S. hospitals and may serve as a pre-pandemic baseline for understanding contextual factors related to digital maturity. The high percentage of hospitals at Stage 0 suggests that systemic barriers, such as insufficient financial incentives, limited digital workforce capacity, fragmented policy mandates, and competing demands for care, may have impeded hospitals’ progress toward digital transformation. These pre-pandemic insights create an opportunity to further investigate the challenges faced by populations with high social disparities seeking care in hospital settings. Research that analyzes the link between digital maturity and hospitals serving populations with high social disparities within the context of pre- and post-pandemic changes in U.S. hospitals would allow for a more complete examination of equitable outcomes among these hospitals. In addition, future studies could examine how digital maturity relates to patient health outcomes, such as quality of care, safety events, or clinical effectiveness, to better understand the impact of digital transformation on clinical performance.

The results of this study are consistent with evidence of the impact of the HITECH Act of 2009, which provided financial incentives to U.S. hospitals to adopt and implement EMRs with the goal of improving quality and safety of healthcare delivery (55, 56). Over the life of the HITECH Act, 98% of U.S. hospitals participated in the program and received $21.8 billion USD in federal incentives, and 60% of eligible office-based providers participated in the program and received $16.2 billion USD in federal incentives. Often referred to as the “meaningful use” policy, it defined success through the implementation of required digital processes such as data collection, adoption of secure email, and tracking the volume of messages, resulting in what some described as a “check the box” compliance program (55). Although the HITECH Act helped accelerate the adoption of basic EMR functions (57), its significant limitations included the dominant focus on process requirements that were disconnected from workflows and clinical best practices, and the absence of outcomes requirements that can be achieved with interoperable data sharing (56, 57). Research has documented the policy's failure to stimulate adoption of advanced EMR functions such as CPOE and data-driven decision-making that support digital maturity progress such as interoperability and health information exchange (56–58). The results of this study support existing evidence that the HITECH Act accelerated EMR adoption across U.S. health systems and demonstrate the critical role of national-level policy to advance digital maturity across state and local jurisdictions (56). However, empirical research on the performance outcomes of EMR adoption, such as equitable access to care, has been limited and may be influenced by the degree to which advanced EMR functionality has been adopted and scaled across hospital organizations (59). The study period also predates later federal interoperability policy developments, including implementation of provisions of the 21st Century Cures Act and the 2020 ONC interoperability rules, which may have influenced hospital interoperability and digital maturity after the study period (61, 62). National policy makers may have an important opportunity to build upon the achievements of the HITECH Act to incentivize progress toward advanced digital maturity functions. In this study, critical-access status was not significantly associated with digital maturity, which suggests that designation alone may not explain variation in digital maturity advancement. Future research that examines factors associated with limited advancement of digital maturity among hospitals will be important to inform evidence-based policy frameworks that support acceleration of digital maturity, particularly among hospitals serving highly vulnerable patient populations.

Examination of MCLs and the burden of UCC among hospitals with low vs. advanced digital maturity revealed no significant difference in MCLs. When we further examined the burden of UCC using its constituent measures, BD and CC, the relationship between CC and digital maturity was conditional on hospital ownership. Among nonprofit hospitals, higher charity-care burden was associated with a lower probability of low digital maturity and higher probabilities of both medium and high digital maturity. Among proprietary hospitals, higher charity-care burden was associated with a lower probability of low digital maturity and a higher probability of medium digital maturity, but not a significantly higher probability of high digital maturity. Among government hospitals, charity-care burden was not significantly associated with digital maturity. This finding may reflect differences in mission, administrative and reporting infrastructure, data systems, financial capacity, or organizational strategy across ownership types. Nonprofit hospitals with higher charity-care burden may be more likely to combine charity care provision with investments in digital maturity, whereas proprietary hospitals with higher charity-care burden may show some movement away from underdeveloped digital maturity without necessarily achieving advanced digital maturity.

The rates of CC relative to hospital ownership have been previously reported, whereby nonprofit hospitals reported a lower ratio of aggregated CC expenses when compared to both government and for-profit hospitals (60). However, digital maturity was not considered as a differentiating factor for hospitals in previous research (28). Our analysis used hospital-level data as opposed to aggregate measures of costing data used in previous research by Bai, G., Yehia, F., and Anderson, G.F. (2020) a limitation acknowledged by the authors. The present findings suggest that ownership structure may be important for understanding how charity-care burden and digital maturity intersect. Specifically, the association between charity care and digital maturity should not be interpreted as uniform across all hospitals, but rather as varying according to ownership type and stage of digital maturity.

Consistent with the expected limitations of using administrative cost and utilization measures as proxies for social disparities, the correlation and PCA analyses indicated that SDI, MCL, and UCC captured related but distinct constructs. SDI was used as the primary area-level measure of social deprivation, whereas MCL, UCC, CC, and BD were treated as hospital-level administrative indicators that may reflect aspects of the populations served as well as hospital service burden, payer mix, uncompensated care, and financial exposure. The weak Pearson correlations between SDI and MCL and between SDI and UCC suggest that these administrative measures should not be interpreted as interchangeable with area-level social deprivation. Rather, MCL and UCC appear to overlap only partially with SDI while capturing additional dimensions of hospital performance and patient population characteristics. These measures should therefore be interpreted as complementary administrative indicators, not as direct substitutes for social deprivation in hospital settings.

Although these findings are confined to considerations for hospitals in the pre-pandemic era, the findings may be interpreted in terms of the functionality of information infrastructure in hospitals in 2018 and 2019 that may now serve as an important baseline for comparative research in the post-pandemic era. Digital infrastructure advances, enabling ease of data access, may offer clinicians greater insights and transparency of data to fully and accurately assess the health needs of patients experiencing high levels of social disparities. More research is needed to examine how digital maturity has progressed since 2020, and how it is associated with charity-care burden across different ownership types. In addition, research that analyzes the contextual factors associated with digital maturity advances is needed to better understand factors that drive or incentivize progress for U.S. hospitals. Factors such as national policy that creates conditions for acceleration, hospital size, population health and social features, regional context, and leadership knowledge of digital maturity's role as a strategy to increase performance warrant further investigation to help produce evidence needed to guide U.S. hospitals. Research that examines hospital capacity to apply informatics research and best practices may provide additional insights into the contextual factors associated with the capacity of U.S. hospitals to advance and strengthen digital maturity as a strategy to increase performance.

As hospitals strive to overcome the many challenges in the post-pandemic era, including global workforce shortages, growing demands for care, and growing complexity of health conditions, senior executives, policy makers, and clinician leaders require contextual evidence to inform policy and decision-making and better understand the impact of digital maturity advances in order to inform strategic decisions to increase performance and quality of care for high-risk populations. Our findings are consistent with, and offer additional strength to previous work (3), which documented a correlation between the SDI and interoperability using the SDI measure based on U.S. census data on social disparities. Results of previous work (3) and this study identify significant digital maturity gaps among hospitals serving populations with high levels of social deprivation. In the current study, differences in digital maturity were most consistently associated with SDI, while charity-care burden showed an ownership-specific pattern, particularly among nonprofit hospitals.

Populations with high levels of social disparities in the U.S. also face high rates of complex health challenges, yet these patients may have reduced access to digitally mature hospitals that support data-driven, coordinated, safe, and high-quality care. As digital maturity among U.S. health systems continues to evolve, hospitals serving populations with the highest social disparities may need to examine how digital maturity advances can help reduce the digital divide rather than deepen inequities in access to coordinated care. More research is needed to examine the impact and role of advanced digital maturity on care delivery strategies in the post-pandemic era to better understand how these advances contribute to meeting the needs of patients with high levels of social disparities and to examine whether digital maturity is associated with equity in access to services and equitable health outcomes for these high-need populations. Advancing digital maturity may be an important strategy to achieve greater quality and safety outcomes for unique patient populations, such as those with high social disparities, by strengthening access to coordinated, data-driven care that is responsive to patients’ life circumstances.

### Limitations

The findings in this study are limited given the use of datasets from 2018 to 2019, and the hospitals in this study may not represent all hospitals in the U.S. The initial EMRAM dataset included 3,355 hospitals with available EMRAM outcome data, of which 2,248 had complete linked EMRAM, SDI, cost, service-burden, and hospital demographic data. The final adjusted analytic model included 2,247 hospitals after excluding one hospital with missing or invalid region information. Hospitals excluded because of incomplete linked data differed from the complete-case sample on several characteristics, including EMRAM maturity, teaching status, urban/rural location, ownership, bed size, and critical-access status. In addition, compared with the remaining hospitals in the CMS-linked hospital file, hospitals in the complete-case EMRAM analytic sample were more likely to be small, rural, critical-access, nonprofit, and governmental hospitals, and less likely to be large, urban, teaching, or proprietary hospitals. Therefore, the complete-case EMRAM analytic sample may not fully represent either all hospitals with available EMRAM records or the broader CMS-linked hospital population, and the findings should be interpreted with caution regarding generalizability.

The 2018–2019 EMRAM dataset predates the many changes in health services delivery that were influenced and accelerated by the pandemic. Social disparities measured by the SDI may also have shifted in the U.S. population since 2018, which is a further limitation of this study's results. In addition, digital technologies have continued to innovate since 2019 and may not be comparable with digital maturity among hospitals today. Finally, although U.S. region was included as an adjustment covariate in the final model, the cross-sectional design does not support causal inference regarding the relationship between social deprivation, hospital characteristics, charity-care burden, and digital maturity.

## Conclusion

Populations with high levels of social disparities in the U.S. also face high rates of complex health challenges, yet these patients may not have access to digitally mature hospitals. In this study, hospitals serving populations in the highest SDI category were more likely to have low EMRAM maturity and less likely to have high EMRAM maturity, even after adjustment for hospital characteristics and U.S. region. As digital maturity among U.S. health systems continues to evolve, hospitals serving populations with the highest social disparities may consider advancing their digital maturity to overcome the digital divide that further challenges these patient populations. Low digital maturity may serve as a structural barrier to equitable care, limiting access to data-driven services and disproportionately impacting populations with high social disparities. Policymakers may consider prioritizing digital maturity advancement as a system-level strategy to improve equity in access and outcomes. Charity-care burden was not uniformly associated with digital maturity across all hospitals; rather, its association differed by ownership type, with the strongest pattern observed among nonprofit hospitals. Building on the achievements of the HITECH Act, national policy that incentivizes digital maturity advances may be well positioned to strengthen quality and safety outcomes for all patients and support more equitable access to coordinated, data-driven care for patient populations with complex needs.

## Data Availability

The data analyzed in this study is subject to the following licenses/restrictions: Owned by an organization (HIMSS). Requests to access these datasets should be directed to anne.snowdon@himss.org.
